# Levator claviculae muscle: a case report

**DOI:** 10.1186/1757-1626-2-6712

**Published:** 2009-05-15

**Authors:** Konstantinos Natsis, Stylianos Apostolidis, Elisavet Nikolaidou, Georgios Noussios, Trifon Totlis, Nikolaos Lazaridis

**Affiliations:** Department of Anatomy, Medical School, Aristotle University of ThessalonikiP.O. Box: 300, 54124 ThessalonikiGreece

## Abstract

In the current study a levator claviculae muscle, found in a 65-year old male cadaver, is presented. We describe the topography and morphology of this accessory muscle, which may be found in 1-3% of the population. Moreover, we discuss the embryologic origin of the muscle along with its clinical importance.

## Introduction

Levator claviculae or cleidocervical muscle is a supernumerary muscle in humans, in contrast to anthropoids and lower mammals where this muscle is found normally [1]. Before the widespread use of computed tomography and magnetic resonance imaging its frequency was underestimated [2]. The last few years the high accuracy of these imaging techniques led to a more frequent recognition of this accessory muscle indicating that it is present in 1-3% of the population [3,4].

Levator claviculae muscle is located in the posterior triangle of the neck. It arises from the transverse processes of the C2-C6 vertebrae and inserts into the middle or lateral third of the clavicle. It may assist thoracic respiration through ribcage's elevation as well as twisting and rotation of the neck. Its innervation is derived from the C2-C5 nerves. The muscle is supplied by the ascending cervical artery [1-5].

In the current study we present a case of a levator claviculae muscle found in a cadaver and we discuss the embryologic origin of the muscle along with its clinical importance.

## Case presentation

**Figure 1.**
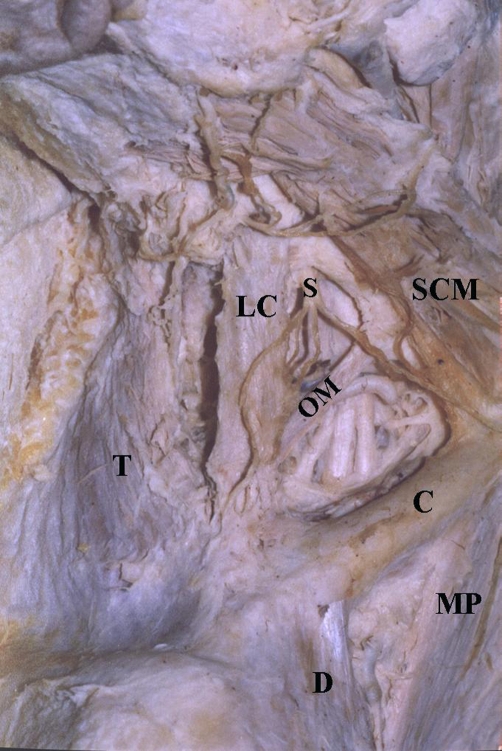
The levator claviculae muscle (LC) found in the right side of the cadaver. T: trapezius muscle; S: supraclavicular nerves; OM: inferior belly of omohyoid muscle; SCM: sternocleidomastoid muscle; C: clavicle; D: deltoid muscle; MP: pectoralis major muscle.

A levator claviculae muscle (Figure 1) was found unilaterally, in the right side of a 65-year old male cadaver, during a routine dissection in our Laboratory. It was a flat and longitudinal muscular slip originating from the transverse processes of the 3^rd^ - 5^th^ cervical vertebrae. Then, it pursued an oblique course, inferiorly, anteriorly and slightly laterally, crossing over the brachial plexus and omohyoid muscle to insert into the acromial extremity of the clavicle. As far as the neurovascular supply of the levator claviculae muscle is concerned, a motor branch of the C4 nerve and the ascending cervical artery supplied this supernumerary muscle.

## Discussion

The embryologic development of the levator claviculae muscle is a controversial issue in the literature and numerous hypotheses have been proposed including its origin from the sternocleidomastoid, trapezius, scalenus anterior or longus colli muscles [5]. Its origin from additional segmentation of the ventrolateral muscle primordia of the neck, proposed by Leon et al. (1987) [5], is accepted by one of the most recent articles on this topic [6] and seems reasonable to us as well.

The levator claviculae muscle is usually an asymptomatic finding in radiologic films [1]. Specifically, it appears as a soft tissue shadow in the posterior triangle of the neck in plain radiograph, computed tomography and magnetic resonance imaging [3,6]. However, it should be differentiated by cysts, haemangiomas, arterial aneurysms, glomus tumors, thrombosed veins, neurofibromas or more often by lymphadenopathy or metastatic lymph nodes [3,4,6]. Indeed, it has been misinterpreted in a computed tomography image as an enlarged lymph node [7]. This is very important since it may have an impact on tumour staging or treatment decisions. This accessory muscle may cause a palpable angular deformity of the clavicle [1]. Recently, Aydog et al. (2007) [8] reported a gymnast with a levator claviculae muscle causing vascular thoracic outlet syndrome.

## Conclusion

The levator claviculae muscle is an anatomical variation with an atavistic character, which means that it is a stump of the development, from our ascendants. It is important not only to anatomists, but also to radiologists, orthopaedics and general surgeons. The variation presented herein appears more frequently than we expect [3], so awareness should be increased during physical examination, in order to avoid further unnecessary diagnostic procedures [6].
